# Supplementary material for: Competing Endogenous RNA Networks in the Epithelial to Mesenchymal Transition in Diffuse-Type of Gastric Cancer
Source: Cancers (Basel). 2020 Sep 24;12(10):2741. doi: 10.3390/cancers12102741 (PMC7598708; doi:10.3390/cancers12102741)
Supplement: Supplementary file 1 [file cancers-12-02741-s001.pdf]

Supplementary Materials: Competing Endogenous RNA Networks in the Epithelial to Mesenchymal Transition in Diffuse-Type of Gastric Cancer

Natalia Landeros, Pablo M. Santoro, Gonzalo Carrasco-Avino and Alejandro H. Corvalan

Table S1. Predicted and validated ceRNA networks based on complementary sequences between lncRNAs, miRNAs and mRNAs associated with EMT process.

lncRNAs	miRNAs	mRNAs associated with EMT
CASC15, DNM3OS, HOTAIR, MAGI2-AS3, SNHG7, TUG1, ZFAS1	miR-9	E-CADHERIN
MNX1-AS1, SNHG7, UCA1, XIST, ZFAS1	miR-15a	TWIST
AFAP1-AS1, DNM3OS, LINC00941, NEAT1, SNHG20, TPT1-AS1	miR-16-1	TWIST
AFAP1-AS1, LINC00941, MNX1-AS1, SNHG7, TUG1, **H19***	miR-22	SNAIL
AFAP1-AS1, CASC15, HOTAIR, LINC00261, MAGI2-AS3, MALAT1, **PVT1**, TPT1-AS1	miR-30a	SNAIL
AFAP1-AS1, **CASC15**, DNM3OS, LINC00261	miR-33a	ZEB, SLUG, SNAIL
AFAP1-AS1, CASC15, HOTAIR, LINC00261, MNX1-AS1, NEAT1, **SNHG7**, TUG1, XIST	miR-34a	SNAIL
DNM3OS, **SNHG6**, SNHG7, TUG1, **XIST**	miR-101	ZEB
AFAP1-AS1, LINC00261, TUG1	miR-124	SLUG
CASC15, DNM3OS, **H19**, **MAGI2-AS3**, MALAT1, MEG3, NEAT1, SNHG20, XIST, ZFAS1	miR-141	ZEB
AFAP1-AS1, DNM3OS, MAGI2-AS3, MALAT1, MEG3, NEAT1, PVT1, ZFAS1	miR-145	ZEB
AFAP1-AS1, CASC15, H19, MNX1-AS1, PVT1, SNHG20, SNHG7, TPT1-AS1, **ZEB1-AS1**	miR-149	ZEB
AFAP1-AS1, DNM3OS, H19, MAGI2-AS3, MEG3, NEAT1, SNHG20, SNHG6, TPT1-AS1, UCA1, XIST	miR-153	SNAIL
AFAP1-AS1, CASC15, DNM3OS, H19, HOTAIR, LINC00261, SNHG6, TUG1, UCA1, XIST	miR-186	TWIST
AFAP1-AS1, CASC15, ZFAS1	miR-195	SNAIL
AFAP1-AS1, MAGI2-AS3, MALAT1, MEG3, SNHG7, TUG1, ZFAS1	miR-199a	E-CADHERIN
AFAP1-AS1, LINC00261, **MAGI2-AS3**, NEAT1, PVT1, TPT1-AS1, TUG1, UCA1, XIST	miR-200	ZEB
No prediction	miR-203	ZEB, SLUG
AFAP1-AS1, DNM3OS, HOTAIR, LINC00941, MAGI2-AS3, ZEB1-AS1	miR-205	ZEB
No prediction	miR-217	E-CADHERIN
AFAP1-AS1, H19, SNHG7, XIST	miR-338	ZEB
No prediction	miR-381	ZEB, TWIST
AFAP1-AS1, H19, SNHG20, TPT1-AS1	miR-491	SNAIL
AFAP1-AS1, CASC15, DNM3OS, PVT1	miR-495	TWIST
AFAP1-AS1, DNM3OS, TUG1	miR-506	ZEB, SLUG
CASC15, LINC00261, PVT1, TPT1-AS1	miR-519d	TWIST
No prediction	miR-544a	E-CADHERIN
AFAP1-AS1, LINC00941	miR-574	ZEB
AFAP1-AS1, DNM3OS, LINC00261, NEAT1, **RP11-789C1.1**	miR-5003	E-CADHERIN

Bold letters denote validated ceRNA networks.
